# The mediating effects of coping style and resilience on the relationship between parenting style and academic procrastination among Chinese undergraduate nursing students: a cross-sectional study

**DOI:** 10.1186/s12912-022-01140-5

**Published:** 2022-12-10

**Authors:** Haitao Huang, Yueming Ding, Yipei Liang, Yiming Zhang, Qianwen Peng, Xiao Wan, Chaoran Chen

**Affiliations:** 1 Institute of Nursing and Health, School of Nursing and Health, Henan University, Kaifeng, People’s Republic of China; 2 School of Business, Henan University, Kaifeng, People’s Republic of China

**Keywords:** Coping, Resilience, Parenting, Procrastination, Nursing students

## Abstract

**Background:**

How to kindle the learning enthusiasm of nursing students and reduce the incidence of academic procrastination is an important factor in reducing student attrition and improving the quality of nursing education.

**Objectives:**

To investigate the mediating role of coping style and resilience on the association between parenting styles and academic procrastination among nursing undergraduates.

**Methods:**

A cross-sectional study of 683 nursing undergraduates was conducted in China from March to May 2022. Parenting styles, coping style, resilience, and academic procrastination were measured using questionnaires. Descriptive analysis, Pearson’s correlation analysis and Hayes' PROCESS Macro in SPSS 25.0 were used to test the model.

**Results:**

Positive parenting style had a significantly direct effect on academic procrastination and through three significantly indirect pathways: (1) through positive coping style (B =  − 0.048, 95% CI: − 0.074 to − 0.025), accounting for 14.71% of the total effect; (2) through negative coping style (B =  − 0.044, 95% CI: − 0.071 to − 0.021), accounting for 13.64% of the total effect; and (3) through resilience (B =  − 0.074, 95% CI: − 0.107 to − 0.044), accounting for 22.82% of the total effect. Moreover, negative parenting style had a significantly direct effect on academic procrastination and through two significantly indirect pathways: (1) through negative coping style (B = 0.056, 95% CI: 0.032 to 0.086), accounting for 21.73% of the total effect, and (2) through resilience (B = 0.028, 95% CI: 0.004 to 0.055), accounting for 10.93% of the total effect.

**Conclusions:**

Intervention measures to reduce the academic procrastination of nursing undergraduates should include the evaluations of coping styles and resilience of nursing students and cultivation strategies to promote their positive coping styles and resilience.

## Introduction

Procrastination refers to the nonadaptive behaviour in which people involuntarily postpone a predetermined plan without a clear reason [1, 2]. Academic procrastination is a form of procrastination in school situations and is related to the fulfilment of studying tasks. Academic procrastination is common among medical college students, with approximately 13.8% to 49.9% of medical students reporting procrastination on learning tasks [3, 4]. Studies have shown that academic procrastination not only leads to a decline in school achievement and negatively affects college students’ learning attitudes, but also leads to negative emotions such as depression and anxiety and can even lead to suicide [5–10]. In addition, academic procrastination can also drain students and prevent them from learning more nursing knowledge and skills [11], which is detrimental to training more nurses and improving the quality of nursing education around the world. At present, growing demand for nursing and increasing global difficulties in recruiting and retaining nurses are undermining nursing outcomes worldwide [12, 13]. Therefore, it is critical to cultivate more professional and enthusiastic nursing students. Thus, it is essential for educators to investigate the risk factors and the mechanisms associated with academic procrastination among nursing students because this information may help to develop coping and prevention strategies that can help to train more high-quality nursing students around the world.

## Background

### Parenting style and academic procrastination

According to Bronfenbrenner's ecological systems theory, family, as a microscopic system, has the most direct influence on individuals [14]. As a stable style of parents in the process of raising children, parenting style is the synthesis of parenting attitudes, concepts and behaviour, reflecting the essence of the parent‒child relationship [15, 16]. Different parenting styles may lead to different coping behaviours [17, 18]. For example, researchers found a significant positive relationship between procrastination and negative parenting [19] and a significant negative relationship between procrastination and positive parenting [20]. Milgram et al. explored the association between college students' procrastination and parenting style and found that autocratic mothers would lead to children's procrastination in life, while autocratic fathers would lead to children's academic procrastination [21]. In addition, studies with Chinese participants have found similar results [22]. Previous studies have documented a link between parenting style and procrastination; however, those studies emphasized college students and rarely involved nursing undergraduates [23, 24]. In China, nursing undergraduates are playing an increasingly important role in nursing education [25]. Data surveys from China show that nurses with advanced diplomas or bachelor's degrees are the most needed workforce at all levels of health care (including tertiary and secondary hospitals) and in the primary care sector (i.e., community health centres, nursing homes or health recovery centre) [25, 26]. In consideration of past results, it is necessary to investigate the association between parenting style and academic procrastination and to gain some understanding of the underlying mechanism of the association.

### The potential mediating effect of coping style

Coping is an individual’s cognitive and behavioural effort to reduce the negative effects of stress and is usually divided into a positive coping style and a negative coping style [27]. Studies have found that the parenting style of an individual will have a significant impact on how individuals respond to stress [28]. As parental accept-rejection theory states, individuals who are often rejected by their parents will exhibit more defensive, hostile and aggressive behaviours in any situation [29]. In contrast,, when parents adopt a positive approach way to raising children, their children are more likely to adopt a positive coping style to solve problems when they experience difficulties [30]. Moreover, many studies have shown that negative coping styles lead to negative outcomes, while positive coping styles, such as positive cognition and seeking support, lead to positive outcomes [31, 32]. Combined with the above views, it is reasonable for us to regard coping style as a mediator between parenting style and academic procrastination.

### The potential mediating role of resilience

Block defines resilience as “an individual’s behavioural tendency to adapt to changing circumstances and the ability to recover from stressful situations” [33, 34]. As an individual stress coping resource, resilience can effectively resist the negative effects of stress [35]. Some studies have pointed out that parenting style is a crucial environment for the formation of adolescents' resilience and that a good parenting style can promote an individual's resilience [36]. It has been found that positive parenting is a favourable factor for adolescents' resilience, while negative parenting is a risk factor for resilience [37, 38]. Furthermore, studies have also indicated that resilience is associated with academic procrastination, and people with high a level of resilience have a lower risk of academic procrastination [39].

### Aims

**Fig. 1:**
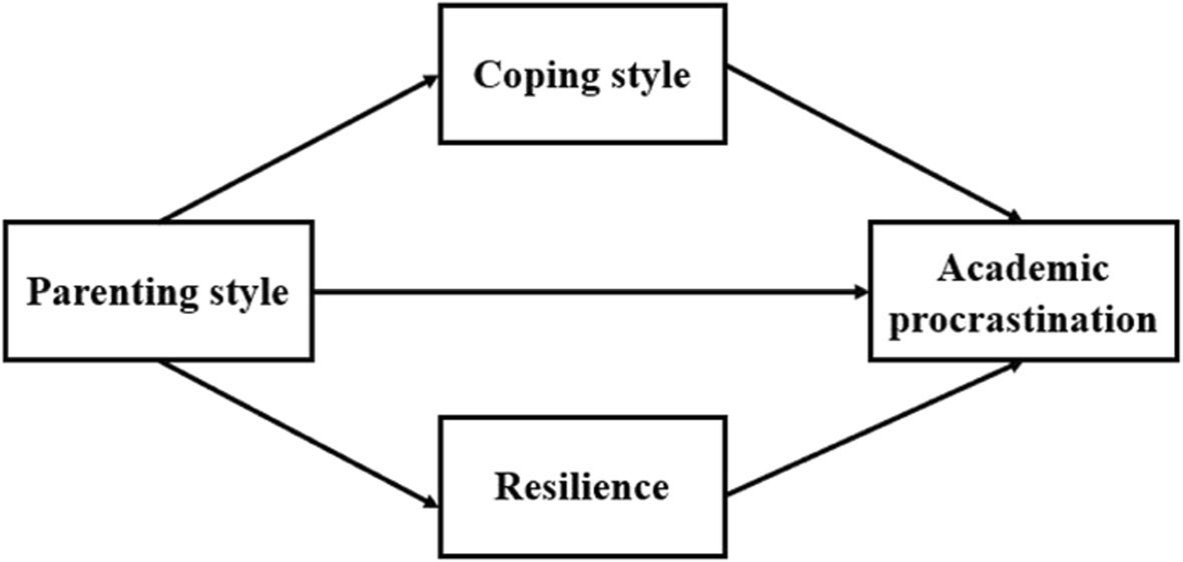
The proposed theoretical mediation model

Based on the above findings, we propose that coping style and resilience may play a mediating variable in the association between parenting style and academic procrastination among undergraduate nursing students. Thus, we investigate the following hypotheses (Fig. 1):

- Hypothesis 1: Parenting style can directly affect the academic procrastination of nursing undergraduates.
- Hypothesis 2: Coping style plays a mediating role in the relationship between parenting style and the academic procrastination of nursing undergraduates.
- Hypothesis 3: Resilience plays a mediating role in the relationship between parenting style and the academic procrastination of nursing undergraduates.

## Methods

### Design

A cross-sectional survey was conducted from March to May 2022.

### Participants

A convenience sampling method was used to recruit nursing undergraduates from two undergraduate universities in Henan, PR China from March to May 2022. The participants met the following inclusion criteria: (1) full-time nursing undergraduates in Grade1, 2 and 3; and (2) knowing the purpose of the research and volunteering to participate in the research. The exclusion criteria were students who did not complete all questionnaires for various reasons. Equation *N* = 4Uα^2^S2/δ^2^ was used to calculate the sample size [40]. S = 0.51 is calculated from the presurvey, the allowable error δ is set to 0.1, and α is set to 0.05, so *N* = 4 × 1.96^2^ × 0.51^2^/0.1^2^ ≈400. Considering the sampling error and possibility of invalid questionnaires, we distributed a total of 687 questionnaires. Finally, after 4 unqualified questionnaires were deleted, 683 results were included in the analysis. In addition, Bentler and Chou [41] proposed that the sample size should be more than 10 times the observed variables; hence, a sample size of 683 met the requirement for testing the hypothesized models.

### Data collection and ethical considerations

Before sampling, we discussed the contents and procedures of the questionnaire and explained the purpose of our study with the psychological services departments of each university. Investigators will began distributing paper questionnaires to students as they gathered in a classroom (approximately 50 students at a time). The questionnaire required approximately 15–20 min of completion time. The participants were not given any incentive or inducement throughout the test. Furthermore, the participants were told that their answers to the questionnaire would be anonymous and confidential and that the data collected would only be used for academic study. This study has been reviewed and approved by the Institutional Review Board of Henan Provincial Key Laboratory of Psychology and Behaviour (reference: 20,220,107,001), and was carried out in accordance with the Declaration of Helsinki.

### Instruments

#### Demographic information

A demographic questionnaire assessed the participants’ characteristics including age, gender, and home location.

#### Chinese version of the parenting bonding instrument (PBI)

The Parenting Bonding Instrument was compiled by Parker [42] and modified by Yang [43]. The scale consists of 46 items, including two subquestionnaires, the Mother version (PBI-M) and the Father version (PBI-F), which both contain 23 items. Each subscale includes three dimensions: care, control and encouragement of autonomy, in which care and encouragement of autonomy are categorised into positive parenting style, and control is categorised into negative parenting style. The scale adopts a 4-point Likert scale (1 to 4 indicate very inconsistent to very consistent, respectively). The revised Chinese version of the PBI has good internal consistency. In this study, the overall Cronbach's α was 0.873, and the Cronbach's α of the positive parenting style dimension and negative parenting style dimension were 0.940 and 0.842, respectively.

#### Short coping style scale (SCSS)

The questionnaire was developed by Xie according to the characteristics of Chinese people [44]. It was composed of two dimensions of positive coping and negative coping, with a total of 20 items. A 4-point Likert scale was used in the questionnaire (1 = not taking, 4 = often taking). Positive coping consists of 12 items, which mainly describe some characteristics of positive coping, such as "try to see the good side of things"; negative coping consists of eight items, focusing on the characteristics of negative coping, such as "imagining that some kind of miracle might happen to change the status quo." In the current study, the overall Cronbach's α was 0.812.The Cronbach's α values of the two subscales were 0.805 and 0.742, respectively.

#### Connor-Davidson resilience scale (CD-RISC)

The CD-RISC was developed by Connor and Davidson in 2003; it which includes 25 items and is divided into five dimensions: tenacity, tolerance of negative effects, positive acceptance of change, control and spiritual influences [45]. The Chinese version of the CD-RISC was revised by Yu [46], retaining 25 items of the original scale and adjusting it to three dimensions: tenacity, strength and optimization; 5-point Likert scales were used, and each item was assigned according to the degree of conformity with the participants’ own situation (0 = never, 4 = almost always). The of CD-RISC (Chinese version) was tested in the general population in China (Cronbach’s α = 0.91) [46]. The Cronbach’s α of the CD-RISC in the current study was 0.91.

#### Aitken procrastination inventory (API)

The API is a self-assessment scale developed by Aitken in 1982 to evaluate the long-term persistent academic procrastination of college students [47]. It has a total of 19 items and has been proven to have good internal consistency in the Chinese context [48]. A 5-point Likert scale was were used, with “1” meaning “completely inconsistent” and “4” meaning “completely consistent”. Items 2, 4, 7, 11, 12, 14, 16, 17, and 18 were inversely scored. Sample items include “I always start things at the last minute.” A high score indicated a higher degree of academic procrastination. The Cronbach's α in the current study was 0.818.

### Statistical analyses

All the data were analysed using IBM SPSS statistics 25.0 (IBM SPSS Statistics for Windows, IBM Corp., Armonk, NY, United States). The demographic characteristics of the participants were represented by descriptive statistics. Continuous variables were calculated from the mean and standard deviation, and intermittent variables were calculated over percentage and frequency. Normality testing was performed on continuous variables using the Kolmogorov‒Smirnov test. We used Pearson correlation analysis to explore the relationship among parenting style, coping style, resilience, and academic procrastination. Harman's single-factor test was used to evaluate the common method bias derived from self-reported data [49]. The mediation effect was tested by PROCESS 4.1 (Model 4) developed by Hayes [50]. In addition, we used the 5000 resample bootstrapping method with a 95% CI to test the model。Gender, age, home location, grade and family structure were controlled as covariate variables in the model. It is assumed that the 95% CI does not contain zero, indicating that the effect is statistically significant. The report of this study is strictly in accordance with the STROBE Statement [51].

### Validity and reliability/ rigour

First, all the instruments used in the study have been adjusted and verified for Chinese culture and have good validity and reliability. In addition, before the formal investigation, all investigators were trained on registration, checking the completeness of questionnaires, and the ethical tenets of conducting research. To reduce the risk of self-reported bias, the identities of all the participants were kept strictly confidential. Finally, to ensure the rigor and accuracy of the statistical analysis, we invited a statistics professor to examine the data processing.

## Results

### Common method biases tests

Harman's single-factor test extracted 29 factors with eigenvalues greater than 1. The first factor explained 15.056% of the total variance, which is below the recommended threshold of 40% [52]. This suggests that common method bias is unlikely to confuse the interpretation of the data analysis results.

### Participants' characteristics

As shown in Table 1, a total of 683 nursing undergraduates effectively participated in the survey, including 152 males (22.3%) and 531 females (77.7%). The age of the participants ranged from 17 to 24 years old (mean = 19.84, SD = 1.19). A total of 36.7% of the participants came from towns, while the remaining 63.3% came from the villages. A total of 42.8% of the participants were freshmen, 34.1% were sophomores and 22.8% were juniors. Finally, 14.1% of the participants were only children, and the vast majority (85.9%) of participants came from families with multiple children.

**Table 1:** Demographic characteristics of undergraduate nursing students. (*N* = 683)

Variable		*N* = 683	%
Gender, *n* (%)			
	Male	152	22.3
	Female	531	77.7
Age, M (SD)		19.84(1.19)	
Grade			
	Grade 1	294	43.1
	Grade 2	233	34.1
	Grade 3	156	22.8
Home Location			
	Town	251	36.7
	Village	432	63.3
Only child in family			
	Yes	96	14.1
	No	587	85.9

### Descriptive analysis and correlations between overall variables

Means, standard deviations (SD), and Pearson correlations of each variable are shown in Table 2. The results show that positive parenting style was significantly correlated with positive coping style (*r* = 0.203, *p* < 0.01), negative coping style (*r* = -0.155, *p* < 0.01) and resilience (r = 0.264, *p* < 0.01) and was significantly negatively correlated with academic procrastination (*r* = -0.289, *p* < 0.01). Moreover, negative parenting style was significantly correlated with negative coping style (*r* = 0.213, *p* < 0.01), resilience (*r* =—0.078, *p* < 0.01) and academic procrastination (*r* = 0.243, *p* < 0.01). Resilience was negatively correlated with academic procrastination (*r* =—0.293, *p* < 0.01). Positive coping style and negative coping style had a significant negative correlation and positive correlation with academic procrastination respectively (*r* =—0.260, *p* < 0.01; *r* = 0.283, *p* < 0.01).

**Table 2:** Means, standard deviations, and correlations of the study variables (*N* = 683)

Variables	Mean ± SD	1	2	3	4	5	6	7	8	9	10	11
PSC	2.16 ± 0.51	1										
PSCL	0.78 ± 0.48	-.320**	1	c								
PSEA	2.19 ± 0.50	.524**	-.456**	1								
PPS	2.18 ± 0.45	.941**	-.416**	.781**	1							
PCS	3.02 ± 0.43	.189**	-0.065	.161**	.203**	1						
NCS	2.38 ± 0.55	-.160**	.213**	-.096*	-.155**	0.044	1					
Tenacity	3.37 ± 0.55	.223**	-0.053	.179**	.234**	.525**	-0.06	1				
Strength	3.60 ± 0.53	.251**	-.131**	.215**	.270**	.550**	-.093*	.755**	1			
Optimization	3.37 ± 0.56	.182**	-0.009	.123**	.182**	.438**	0.068	.564**	.621**	1		
Resilience	3.45 ± 0.49	.250**	-.078*	.202**	.264**	.577**	-0.055	.949**	.901**	.727**	1	
AP	2.55 ± 0.50	-.274**	.243**	-.223**	-.289**	-.260**	.283**	-.265**	-.317**	-.154**	-.293**	1

*Abbreviations: PSC* Parenting style-care, *PSCL* Parenting style-control, *PSEA* Parenting style- encourage autonomy, *PPS* Positive parenting style, *PCS* Positive coping style, *NCS* Negative coping style, *AP* Academic procrastination

^*^P < 0.05

^**^ *P* < 0.01

### Testing the mediation effect of positive parenting style on academic procrastination

**Fig. 2:**
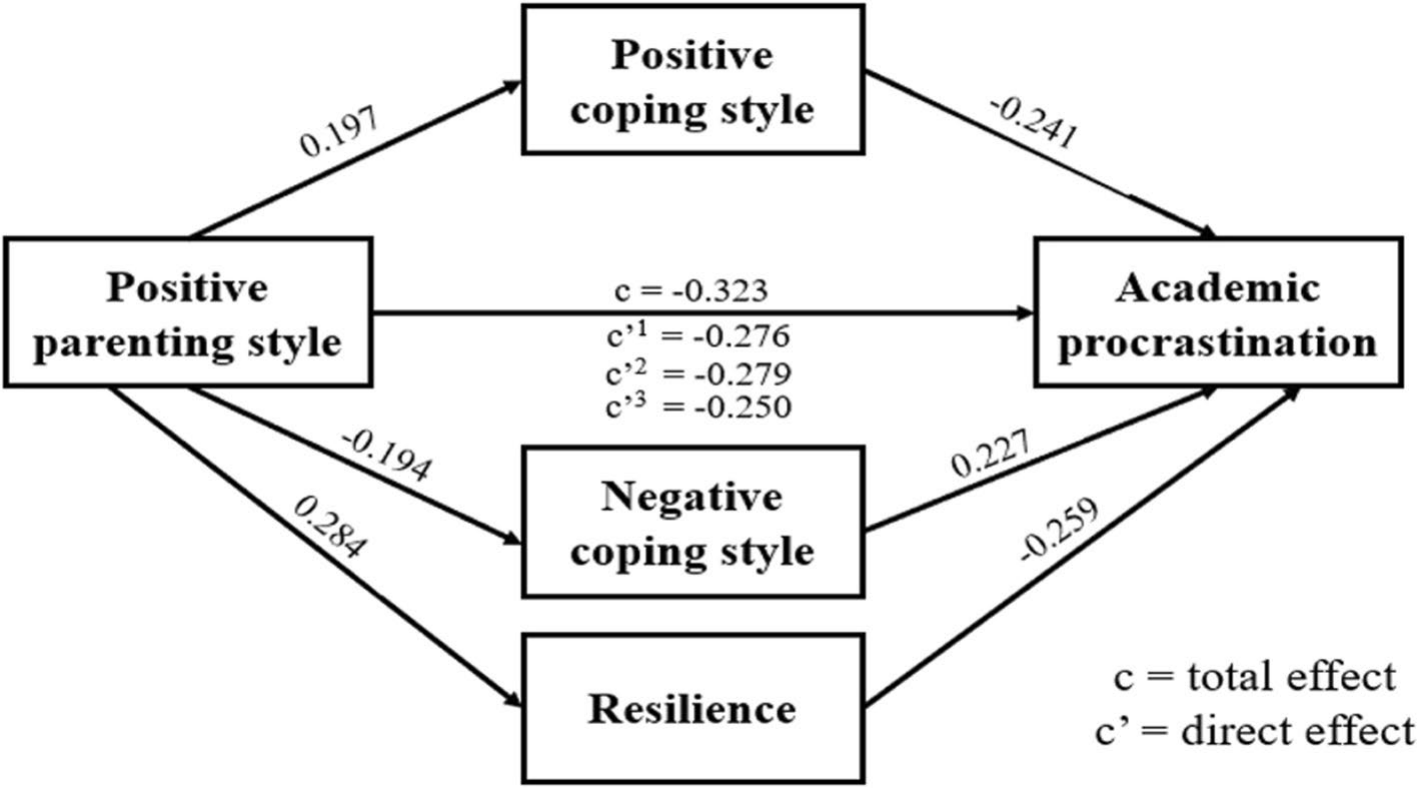
The mediating effect of coping style and resilience between positive parenting style and academic procrastination

As shown in Fig. 2, positive parenting style was negatively related to academic procrastination (B = -0.323, *p* < 0.001). After adding the mediating variables positive coping style, negative coping style and resilience respectively, the negative relation between positive parenting style and academic procrastination was still significant (positive coping style: B = -0.276, *p* < 0.001; negative coping style: B = -0.250, *p* < 0.001; resilience: B = -0.279, *p* < 0.001). Positive parenting style was also positively related to positive coping style (B = 0.197, *p* < 0.001), and positive coping style was negatively related to academic procrastination (B = -0.241, *p* < 0.001). Positive parenting style was also negatively related to negative coping style (B = -0.194, *p* < 0.001), and negative coping style was positively related to academic procrastination (B = 0.227, *p* < 0.001). Moreover, positive parenting style was positively related to resilience (B = 0.284, *p* < 0.001), and resilience was negatively related to academic procrastination (B = -0.259, *p* < 0.001). This showed that positive parenting style was not only directly associated with academic procrastination, but also indirectly associated with academic procrastination through positive coping style, negative coping style and resilience, which played a partial mediating role. Table 3 shows the ratio of the direct effect and the indirect effect to total effect respectively.

**Table 3:** Total, direct and indirect effects of Parenting Style on academic procrastination

Path	Effect	Effect	Boot SE	Boot LLCI	Boot ULCI	Relative mediation effect
PPS-PCS-AP	Direct effect	-0.276	0.043	-0.358	-0.190	85.39%
	Indirect effect	-0.048	0.013	-0.074	-0.025	14.71%
	Total effect	-0.323	0.041	-0.404	-0.242	
PPS-NCS-AP	Direct effect	-0.279	0.042	-0.360	-0.195	86.36%
	Indirect effect	-0.044	0.013	-0.071	-0.021	13.64%
	Total effect	-0.323	0.041	-0.404	-0.242	
PPS- Resilience -AP	Direct effect	-0.250	0.043	-0.331	-0.166	77.28%
	Indirect effect	-0.074	0.016	-0.107	-0.044	22.82%
	Total effect	-0.323	0.041	-0.404	-0.242	
NPS-NCS-AP	Direct effect	0.201	0.039	0.124	0.276	78.23%
	Indirect effect	0.056	0.014	0.032	0.086	21.73%
	Total effect	0.257	0.040	0.179	0.335	
NPS-Resilience-AP	Direct effect	0.229	0.039	0.154	0.304	89.07%
	Indirect effect	0.028	0.013	0.004	0.055	10.93%
	Total effect	0.257	0.040	0.179	0.335	

*Abbreviations: PPS* Positive parenting style, *PCS* Positive coping style, *AP* Academic procrastination, *NCS*, Negative coping style, *NPS* Negative parenting style, *Boot LLCI* the lower limit confidence interval of effects estimated by Bootstrap Method, *Boot SE* the standard error of effects estimated by Bootstrap method, *Boot ULCI* the upper limit confidence interval of effects estimated by Bootstrap method

### Testing the mediation effect of negative parenting style on academic procrastination

**Fig. 3:**
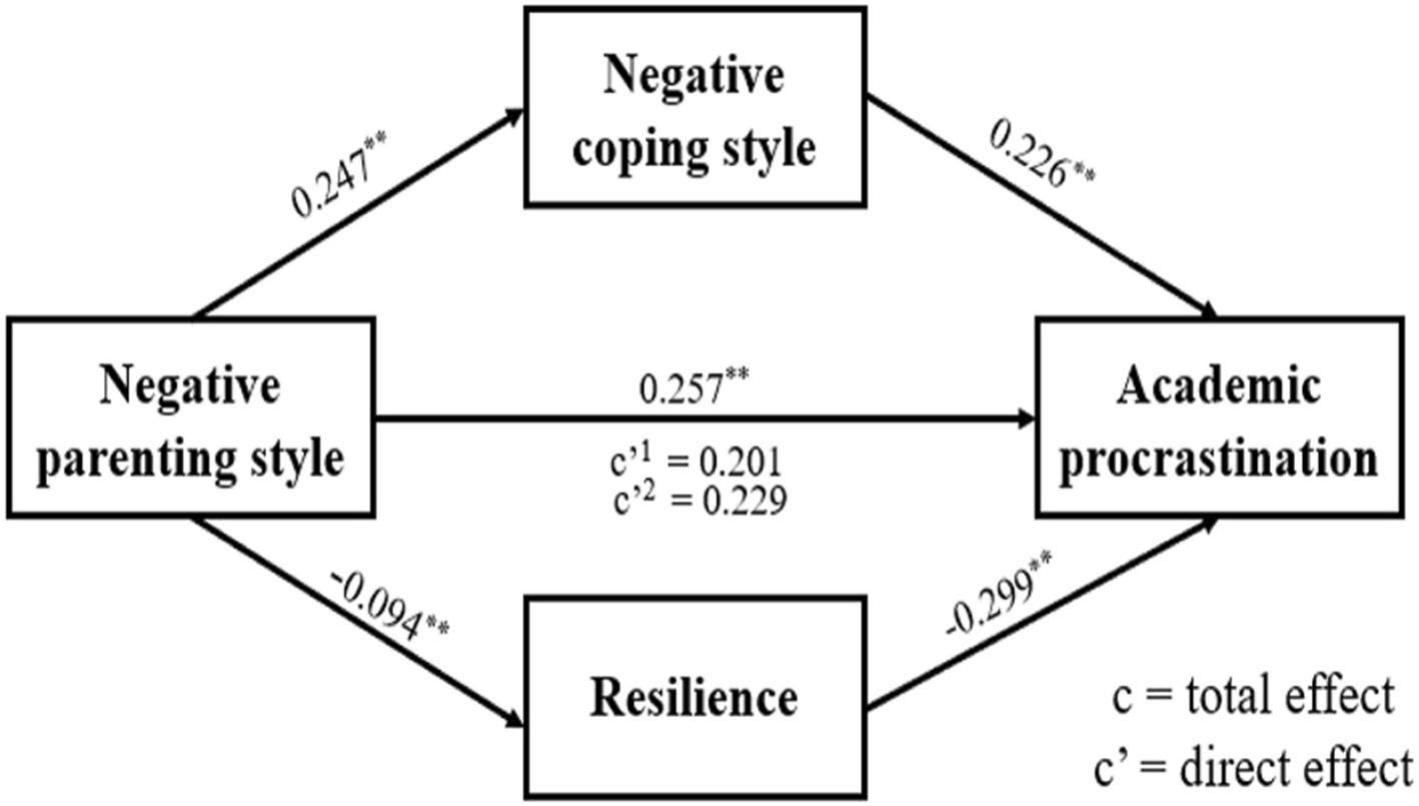
The mediating effect of negative coping style and resilience between negative parenting style and academic procrastination

Figure 3 shows that negative parenting style was negatively related to academic procrastination (B = 0.257, *p* < 0.001). After adding the mediating variables of negative coping style and resilience respectively, the positive relation between negative parenting style and academic procrastination was still significant (coping style: B = 0.201,* p* < 0.001; resilience: B = 0.229, *p* < 0.001). Negative parenting style was positively related to negative coping style (B = 0.247, *p* < 0.001), and negative coping style was positively related to academic procrastination (B = 0.226, *p* < 0.001). In addition, negative parenting style was also negatively related to resilience (B = -0.094, *p* < 0.001), and resilience was negatively related with academic procrastination (B = -0.299, *p* < 0.001). This shows that negative parenting style was not only directly associated with academic procrastination, but also indirectly associated to academic procrastination through negative coping style and resilience respectively which played a partial mediating role. Table 3 shows the ratio of the direct effect and indirect effect to the total effect respectively.

## Discussion

Our study aimed to explore the mediating roles of coping style and resilience in the association between parenting style and academic procrastination in nursing undergraduates, respectively. We examined the association among parenting style, coping style, resilience and academic procrastination, and the direction in which they are affected.

First, the results showed that positive parenting style and negative parenting style significantly negatively and positively predicted the academic procrastination of nursing students, respectively. The results support Hypothesis 1. This result is consistent with the previous results [22, 53]. Parenting style, as an important part of the family micro system, has an important impact on individual personality, behaviour and attitude [54, 55]. As a behavioural attitude, academic procrastination is also affected by the family environment, especially parenting style. Students living in a warm family environment are more likely to obtain more support and understanding and are more likely to treat difficulties in the learning process with a positive attitude, thus showing a lower level of academic procrastination. In contrast, students who are consistently rejected and punished by their parents find it difficult to obtain adequate understanding and support, are more likely to experience frustration and helplessness, and tend to have negative attitudes towards their studies, which is manifested as academic procrastination [53].

Second, this study found that positive parenting style not only directly negatively affects academic procrastination but also indirectly negatively affects academic procrastination through the mediating effect of positive coping style and negative coping style, respectively. In contrast, a negative parenting style not only directly positively affects academic procrastination, but also indirectly positively influences academic procrastination through the mediating effect of a negative coping style. The results support Hypothesis 2. This indicates that a positive parenting style is beneficial to the formation of a positive coping style among nursing students, and has a weakening effect on negative coping styles, such that they believe that personal ability and effort are closely related to the success or failure at school, so that they can actively engage in learning. However, the negative parenting style easily leads to lead to the formation a negative coping style, which makes them believe that the success or failure of their studies depends on luck, opportunity and other external factors so that they are not willing to actively invest in learning [56]. As mentioned in the introduction, the formation of coping styles is more subject to external environmental factors, among which the family environment is particularly important for individual physical and mental development [17]. Parenting style, as a stable style tendency of parents in the process of raising children, can to some extent determine whether adolescents can better cope with stressful events [57]. Previous studies have shown that individual coping style plays a mediating effect in the association between parenting style and many behavioural outcomes [56, 58]. In our study, coping style has a mediating effect on the influence of parenting style on academic procrastination in nursing undergraduates: it is not only affected by parenting style but also significantly predicts students' academic procrastination level.

Third, in addition to coping style, resilience also partially mediated the influence of parenting style on academic procrastination among nursing undergraduates. Specifically, positive parenting style not only negatively affected academic procrastination directly but also indirectly through the mediation of resilience. While negative parenting style has a direct positive impact on academic procrastination, it also has an indirect positive impact on academic procrastination through the mediating effect of resilience. This finding supports Hypothesis 3. Resilience is an effective resource for coping with stressful situations and reflects individual social adaptability [59]. In previous studies, resilience has generally been discussed as a protective factor of individual psychology and behaviour [60]. Nursing undergraduates will inevitably encounter difficulties and challenges in the learning process. To maintain a positive learning state, they need not only strong internal learning motivation but also good self-control, especially resilience [61]. Nursing students with high resilience are less affected by high stress and more likely to recover as soon as possible, flexibly adjust learning strategies, and actively engage in learning. However, students with poor resilience have a weak ability to resist pressure and cannot actively adjust their learning strategies, so they cannot maintain a low level of academic procrastination [62]. Resilience can effectively reduce the level of academic procrastination because it can improve students' self-esteem and self-efficacy [63] and enhance their psychological endurance and their ability to resist the temptation of short-term interests [63], so that more psychological resources can be put into learning and effectively reduce their learning procrastination level. Cleary [64] suggests that nursing students are in an important stage of development, and resilience is a necessary trait for them to achieve success in learning and practice. Therefore, nursing educators can take a comprehensive approach to improving the resilience of nursing undergraduates through relevant courses, such as individual counseling and group counselling to reduce the risk of academic procrastination.

### Implications for nursing education

The results of this study have important theoretical significance and practical value for improving the academic procrastination of undergraduate nursing students. To reduce the risk of academic procrastination, the following recommendations are made. First, for parents of nursing students, they should provide a positive and democratic parenting style for nursing students. Parents should give their children more affirmation, encouragement and praise and reduce denial, rejection and criticism so that nursing students can feel the love of their parents, which can help them be devoted to their studies with more enthusiasm and thus reduce the incidence of academic procrastination. Nursing educators can provide a good supportive learning environment during their nursing education practice. Nursing students can also be encouraged to actively participate in class and community activities through various means, such as individual or group counselling, to develop their level of resilience and positive coping styles so that they can have the confidence and courage to cope with the difficulties and setbacks they encounter in their studies and lives, thereby reducing the risk of academic procrastination. Third, nursing students themselves should develop good study habits and scientifically improve their time management skills. In addition, appropriate relaxation, such as listening to music or running, may also be a good way to relieve academic stress and reduce academic procrastination when an individual is under academic pressure. Finally, nursing education authorities can regularly invite psychological experts to carry out psychological training, teaching nursing students to cultivate resilience and positive coping skills to improve the learning efficiency of nursing students and reduce the occurrence of procrastination.

### Limitations

The results are of great significance for the implementation of interventions aimed at reducing academic procrastination in nursing students. Although there are some highlights, several limitations must be considered. First, this study is a cross-sectional study, so further longitudinal studies are needed to investigate the causal associations.. Second, the data used in the current research were all self-reported by the participants, which may affect the results through recall bias. Although the deviation of common methods was not found in this study, a variety of data collection methods (such as the combination of self-report and others' report) should be used in future studies to ensure the reliability of our conclusions. Finally, the participants in this study are from only two undergraduate universities, which hinders the generalisability of the conclusion to some extent. Future studies can expand sample sources and explore the differences in results under different cultural backgrounds and educational levels.

## Conclusion

In the context of a global nursing shortage, measures to reduce nursing staff turnover and improve the quality of nursing education, such as reducing academic procrastination among nursing students, are an urgent task. This study found that coping style and resilience not only directly affected academic procrastination in nursing students but also partially mediated the relationship between parenting style and academic procrastination. In view of these findings, it is necessary for nursing educators to develop an academic procrastination improvement strategy suitable for nursing undergraduates.

## Data Availability

The datasets used and/or analysed during the current study are available from the corresponding author on reasonable request.
